# Draft Genome Sequence of *Zobellia* sp. Strain OII3, Isolated from the Coastal Zone of the Baltic Sea

**DOI:** 10.1128/genomeA.00737-17

**Published:** 2017-09-07

**Authors:** Henrik Harms, Anja Poehlein, Andrea Thürmer, Gabriele M. König, Till F. Schäberle

**Affiliations:** a Institute for Pharmaceutical Biology, University of Bonn, Bonn, Germany; b German Center for Infection Research (DZIF) Partner Site Bonn/Cologne, Bonn, Germany; c Department of Genomics and Applied Microbiology and Göttingen Genomics Laboratory, Georg-August University Göttingen, Göttingen, Germany; d Institute for Insect Biotechnology, Justus Liebig University Giessen, Giessen, Germany

## Abstract

*Zobellia* sp. strain OII3 was isolated from a marine environmental sample due to its heterotrophic lifestyle, i.e., using *Escherichia coli* cells as prey. It shows strong agar-lytic activity. The genome was assembled into 41 contigs with a total size of 5.4 Mb, revealing the genetic basis for natural product biosynthesis.

## GENOME ANNOUNCEMENT

The genus *Zobellia* contains Gram-negative marine bacteria that show gliding activity (1). Genome sequences were previously available for two strains, i.e., *Z. galactanivorans* DSM12802 (RefSeq NC_015844) and *Z. uliginosa* (RefSeq NZ_JQMD00000000). *Zobellia* spp. have been isolated from marine material, especially algae, and various algae produce galactans like agar and carrageenan. *Zobellia* spp. seem to be involved in the degradation of algae in the marine environment, and the capability to degrade agar actively is a key feature of this genus, which became a model for studying algal polysaccharide bioconversions (2).

The strain described here was isolated during a bioproject aiming to discover novel compounds with biological activities. It was retrieved from marine sediment collected at the coastal zone of Kappeln, Germany, using *Escherichia coli* cells as prey. The same method was established earlier for terrestrial bacteria, e.g., myxobacteria, which show a high potential for the production of biologically active specialized compounds (3–5). The same strategy proves true for marine samples; in particular, bacteria attached to surfaces of organic material have been isolated in this way. The heterotrophic lifestyle seems to be an advantageous feature for these organisms (5). The ability of *Zobellia* spp. to degrade agar is particularly pronounced and can be observed by every growing culture on agar plates. The colonies sink into the agar due to the degrading activity. Screening of the extract from *Zobellia* sp. strain OII3, grown 7 days in ASW-CY medium (casiton 3 g/liter, yeast extract 1 g/liter,  KCl  0.99 g/liter,  KBr  0.15  g/liter, NaCl  35.22 g/liter,  MgCl × 6  H_2_O 15.92 g/liter, CaCl_2_ × 2 H_2_O, SrCl × 6 H_2_O 0.06 g/liter, Na_2_SO_4_ anhydrous 5.88 g/L, NaHCO_3_ 0.29 g/liter, and H_3_BO_3_ 0.05 g/liter, trace element solution 1 ml/liter, cyanocobalamine stock solution 1 ml/liter; trace element solution: 20 mg ZnCl_2_, 100 mg MnCl_2_ × 4 H_2_O, 10 mg H_3_BO_3_, 10 mg CuSO_4_, 20 mg CoCl_2_, 5 mg SnCl_2_ × 2 H_2_O, 5 mg LiCl, 20 mg KBr, 20 mg KI, 10 mg Na_2_MoO_4_ × 2 H_2_O and 5.2 g Na_2_-EDTA × 2 H_2_O dissolved in 1 liter distilled water; cyanocobalamine stock solution: 0.5 mg dissolved  in 1 ml water), revealed antimicrobial activity against Gram-positive test strains.

A sample was prepared for sequencing by growing the strain aerobically at 30°C in ASW-CY medium for 3 days. Extraction of the genomic DNA was performed by using the GenElute bacterial genomic DNA kit (Sigma Aldrich). The extracted DNA was used to generate Illumina shotgun paired-end sequencing libraries, which were sequenced with a MiSeq instrument and the MiSeq reagent kit version 3, as recommended by the manufacturer (Illumina). Quality filtering using Trimmomatic version 0.36 (6) resulted in 2,211,244 paired-end reads. The assembly was performed with the SPAdes genome assembler software version 3.10.0 (7). The assembly resulted in 41 contigs (>500 bp) and an average coverage of 92.87-fold. The assembly was validated, and the read coverage was determined with QualiMap version 2.1 (8). The resulting draft genome is 5,396,230 bp in length, while the GC content is 42.6%. The sequence was annotated with the Rapid Annotations using Subsystems Technology (RAST) prokaryotic genome annotation server (version 2.0) using standard procedures (9), yielding 4,648 coding sequences. An antiSMASH (10) analysis was performed to identify putative biosynthetic gene clusters. In total, five putative clusters were identified, i.e., those coding for an aryl polyene type III polyketide, resorcinol, two terpenes, and aryl polyene-resorcinol.

The same clusters were found in the closest relative strain, *Z. galactanivorans* DSM12802 (RefSeq NC_015844), which harbored an additional putative type I PKS biosynthetic gene cluster that was not detected by antiSMASH in the strain reported here. The genome of *Zobellia* sp. OII3 will contribute to the identification of the antibiotically active component(s) of this strain.

### Accession number(s).

This whole-genome shotgun project has been deposited at DDBJ/ENA/GenBank under the accession no. MWRZ00000000. The version described in this paper is the first version, MWRZ01000000.
